# Reply to Akdogan et al. Comment on “Öztürk et al. Association Between Fresh Gas Flow Rate and Optic Nerve Sheath Diameter During Laparoscopic Cholecystectomy: A Prospective Observational Study. *J. Clin. Med.* 2026, *15*, 7626”

**DOI:** 10.3390/jcm15218581

**Published:** 2026-11-04

**Authors:** Mervenur Gizem Öztürk, Öznur Demiroluk, Mehmet Timuçin Aydın, Arzu Yıldırım Ar

**Affiliations:** 1 Department of Anesthesiology and Reanimation, Fatih Sultan Mehmet Training and Research Hospital, University of Health Sciences, Istanbul 34752, Türkiye; 2 Department of Anesthesiology and Reanimation, Karakoçan State Hospital, Elazığ 23600, Türkiye; 3 Department of General Surgery, Fatih Sultan Mehmet Training and Research Hospital, University of Health Sciences, Istanbul 34752, Türkiye

We thank Akdogan and colleagues for their valuable comments [1] on our recently published study and for highlighting the importance of remifentanil exposure when interpreting the observed differences in age-adjusted minimum alveolar concentration (MAC). We welcome the opportunity to clarify our anesthetic management protocol and the scope of the available data.

As described in our original article [2], desflurane and remifentanil were titrated according to the same clinical and physiological targets in both groups. However, individual remifentanil infusion rates and cumulative doses were not separately recorded in the study dataset. Therefore, the potential contribution of between-group differences in opioid exposure to the transient MAC differences observed at T2 and T3 cannot be assessed retrospectively.

Fresh gas flow-dependent volatile anesthetic kinetics may also have contributed to these differences. Fresh gas flow rate may influence the delivery of volatile anesthetics into the breathing circuit, changes in inspired concentrations, and the time required for alveolar concentrations to reach their target levels. Given the low blood–gas solubility of desflurane, differences in anesthetic delivery and concentration equilibration associated with fresh gas flow rate may provide a plausible explanation for the transient MAC differences observed at T2 and T3 [3,4].

In this context, Şakar et al. [5] reported lower MAC values at certain time points during low-flow desflurane anesthesia, without a statistically significant between-group difference in total fentanyl consumption. Although differences in anesthetic protocols and the opioids used preclude direct extrapolation of these findings to the mechanism underlying our observations, they support consideration of flow-dependent kinetic effects. Furthermore, remifentanil titration may be influenced by intraoperative responses associated with changes in desflurane delivery; therefore, these two mechanisms should not necessarily be regarded as entirely independent. Accordingly, the transient MAC differences should be interpreted within a framework that considers both potential differences in opioid titration and fresh gas flow-dependent volatile anesthetic kinetics.

We also appreciate the suggestion to report inspired and end-tidal desflurane concentrations. Although these parameters were monitored intraoperatively using the anesthesia workstation, they were not separately recorded in the study dataset because our study was primarily designed to evaluate perioperative changes in optic nerve sheath diameter (ONSD), rather than to characterize the delivery kinetics of volatile anesthetic agents. Nevertheless, we acknowledge that systematic recording of inspired and end-tidal desflurane concentrations, together with remifentanil infusion rates and cumulative doses, could facilitate a more detailed evaluation of the potential mechanisms underlying the observed transient MAC differences.

Importantly, our study did not assume equivalent opioid exposure on the basis of similar bispectral index (BIS) values. The absence of a statistically significant group effect or group-by-time interaction in BIS was reported as an observation regarding BIS-assessed hypnotic depth, rather than as evidence of comparable remifentanil administration between groups. The primary objective of our study was to evaluate perioperative ONSD changes under two different fresh gas flow strategies. MAC and BIS findings were secondary observations describing the anesthetic conditions under which ONSD was assessed.

In this context, the inability to fully elucidate the potential mechanisms underlying the transient MAC differences does not alter the reported statistical results for ONSD, the primary outcome of our study. However, the absence of detailed opioid exposure data and systematically recorded inspired and end-tidal desflurane concentrations limits the extent to which the observed ONSD findings can be attributed specifically to fresh gas flow rate. Accordingly, these findings should be interpreted as associations observed under the two anesthetic management strategies rather than as evidence of an isolated causal effect of fresh gas flow.

We agree that future prospective studies systematically recording opioid exposure and inspired and end-tidal desflurane concentrations could contribute to a more comprehensive assessment of the anesthetic conditions under which ONSD measurements are obtained. In particular, studies incorporating randomized allocation to fresh gas flow groups may help clarify the relative contributions of volatile anesthetic kinetics and opioid titration, and further investigate the relationship between fresh gas flow rate and perioperative ONSD changes.

We again thank Akdogan and colleagues for their constructive comments and for the opportunity to elaborate on the interpretation of our findings.

## Data Availability

No new data were created or analyzed in this study. Data sharing is not applicable to this article.
